# Supplementary material for: Limits of topological protection under local periodic driving
Source: Light Sci Appl. 2019 Jul 10;8:63. doi: 10.1038/s41377-019-0172-8 (PMC6804922; doi:10.1038/s41377-019-0172-8)
Supplement: Supplementary file 1 — Supplemental Material [file 41377_2019_172_MOESM1_ESM.docx]

**Supplementary material: Limits of topological protection under local periodic driving**

Z. Cherpakova,^1,*^C. Jörg,^2,*^C. Dauer,^2^F. Letscher,^2,3^M.Fleischhauer,^2^S. Eggert,^2^S. Linden,^1,+^and G. von Freymann^2,4,±^

*^1^Physikalisches Institut, Universität Bonn, 53115 Bonn, Germany*

*^2^Physics Department and Research Center OPTIMAS, TU Kaiserslautern, 67663 Kaiserslautern, Germany*

*^3^Graduate School Materials Science in Mainz, 67663 Kaiserslautern, Germany*

*^4^Fraunhofer Institute for Industrial Mathematics ITWM, 67663 Kaiserslautern, Germany*

*these authors contributed equally to this work

+ linden@physik.uni-bonn.de

± georg.freymann@physik.uni-kl.de

**Role of the on-site potential for the experiments:**

In case of the DLSPPW waveguides the on-site potential is very small due to strong confinement of SPPs. We have performed the measurements with isolated curved waveguides and estimated *ΔV* to be approximately three times less than the amplitude of coupling variation *ΔJ* even at the highest frequency. Our Fourier-space measurements also confirm that (see Fig. 5 (h)).

In the case of the dielectric waveguides, we have calculated the band structure for the parameters used for the experiments in Fig. 6(a). The result of the calculations is shown below in Fig. S1. It is seen that the only visible effect of the on-site potential variation caused by the curvature of the waveguides is the overall shift of an edge state together with its replicas towards higher energies by *ΔV*. This effect is more pronounced for high frequencies, as *ΔV* scales with *ω*² (see Eq. 19). Due to this shift, one of the replicas hits the bulk bands a bit earlier than the other with increasing frequency. For *ω*/*J*_1_<1.6 we have *ΔV<ΔJ.* However, even for *ω*/*J*_1_=2.2, *ΔV* is still smaller than the bandgap, and *ω* is so large that the first Floquet replicas lie well outside the bulk bands. For the measurements in Fig. 6(b), *ΔV<ΔJ* is valid for all wavelengths used. Therefore, the on-site potential does not change the overall picture qualitatively.


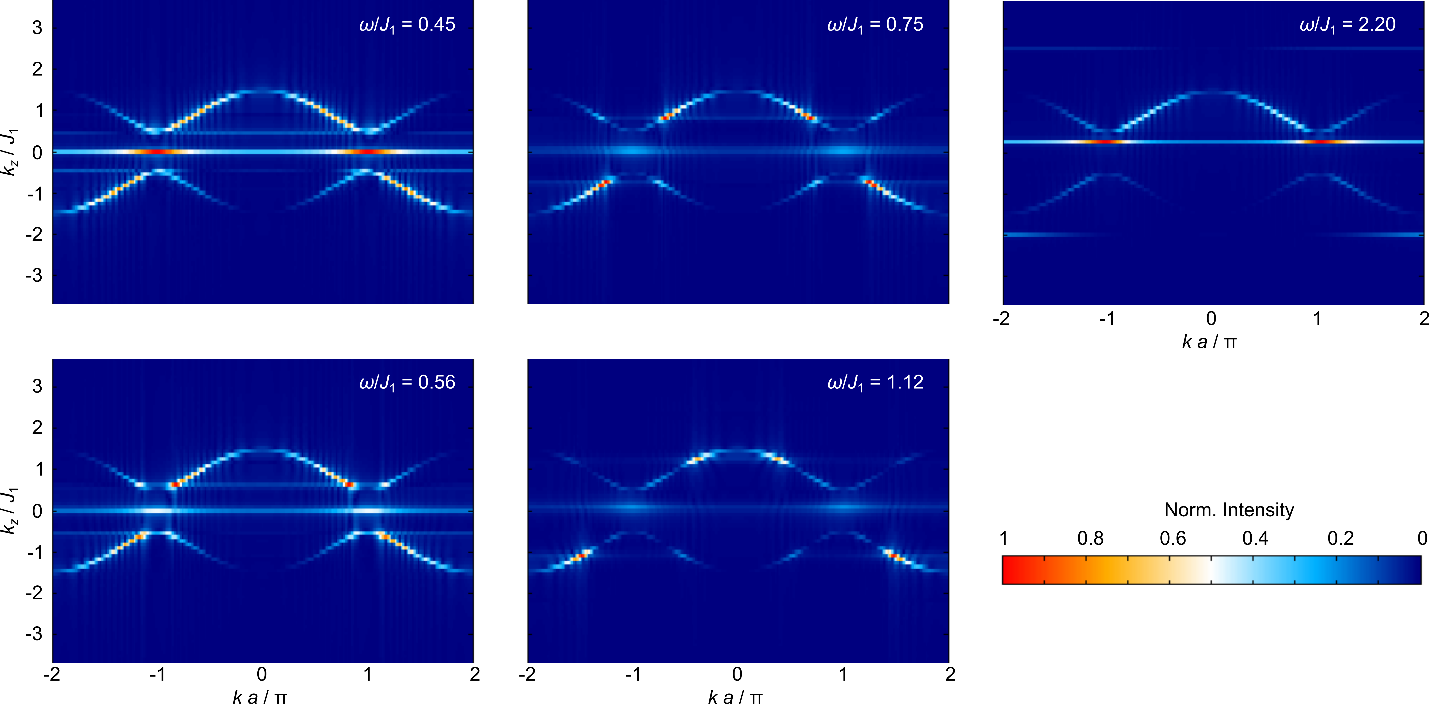


Fig. S1: Numerically calculated band structures for the experimental parameters of Fig. 6(a).
